# *Gnomoniopsis chinensis* (Gnomoniaceae, Diaporthales), a new fungus causing canker of Chinese chestnut in Hebei Province, China

**DOI:** 10.3897/mycokeys.67.51133

**Published:** 2020-05-14

**Authors:** Ning Jiang, Ling-Yu Liang, Cheng-Ming Tian

**Affiliations:** 1 The Key Laboratory for Silviculture and Conservation of the Ministry of Education, Beijing Forestry University, Beijing 100083, China Beijing Forestry University Beijing China

**Keywords:** *Castanea mollissima*, chestnut disease, taxonomy

## Abstract

Chinese chestnut (*Castanea mollissima*) is an important crop tree species in China. However, branch canker and fruit rot are two kinds of severe diseases, which weaken the host and decrease chestnut production. During our investigations into chestnut diseases in China, several fungi have been confirmed as casual agents in previous studies, namely *Aurantiosacculus castaneae*, *Cryphonectria neoparasitica*, *Cry. parasitica*, *Endothia chinensis* and *Gnomoniopsis daii*. In this study, a new canker pathogen is introduced based on morphology, phylogeny and pathogenicity. Typical *Gnomoniopsis* canker sign of wide, orange tendrils emerging from hosts’ glaucous lenticels were obvious on the diseased trees in the field. Symptomatic branches or bark on stems from different chestnut plantations were sampled and isolated, then strains were identified by comparisons of DNA sequence data for the nuclear ribosomal internal transcribed spacer (ITS), partial translation elongation factor-1α (*tef1*) and β-tubulin (*tub2*) gene regions as well as morphological features. As a result, these strains appeared different from any known *Gnomoniopsis* species. Hence, we propose a novel species named *Gnomoniopsis chinensis*. Pathogenicity was further tested using the ex-type strain (CFCC 52286) and another strain (CFCC 52288) on both detached branches and 3-year-old chestnut seedlings. The inoculation results showed that *Gnomoniopsis chinensis* is mildly pathogenic to Chinese chestnut. However, further studies are required to confirm its pathogenicity to the other cultivated *Castanea* species in America, Europe and Japan.

## Introduction

The Chinese chestnut (*Castanea mollissima*), as well as the American chestnut (*C. dentata*), the European chestnut (*C. sativa*) and the Japanese chestnut (*C. crenata*), are known as the four main cultivated sweet chestnut species in the world (Conedera et al. 2004; Yi 2017). In recent studies, several important fungal pathogens have been reported from chestnut trees, including *Aurantiosacculus castaneae*, *Cryphonectria neoparasitica*, *Cry. parasitica*, *Endothia chinensis* and *Gnomoniopsis daii* from *C. mollissima* (Jiang et al. 2018a, 2019b; Jiang and Tian 2019); *Cry. parasitica*, *G. smithogilvyi* (syn. *G. castaneae*), *Phytophthora cinnamomi* and *Sirococcus castaneae* from *C. sativa* (Anagnostakis 1987; Visentin et al. 2012; Shuttleworth et al. 2013; Meyer et al. 2017; Shuttleworth and Guest 2017; Rigling and Prospero 2018; Akilli Şimşek et al. 2019; Lione et al. 2019). In China, *Castanea mollissima* is widely cultivated for its gluten-free, low fat, and cholesterol-free chestnuts (Lu and Guo 2017), but suffering from several fungal diseases (Li et al. 2006; Zhang et al. 2009).

The fungal genus *Gnomoniopsis* (Gnomoniaceae, Diaporthales) includes species all occurring in plant tissues as pathogens, endophytes or saprobes (Danti et al. 2002; Rossman et al. 2007; Walker et al. 2010; Sogonov et al. 2008). Until now, *Gnomoniopsis* species have been found on hosts from three plant families, Fagaceae, Onagraceae and Rosaceae (Sogonov et al. 2008; Walker et al. 2010). Two species occur as pathogens on *Castanea* species (family Fagaceae), i.e. *Gnomoniopsis smithogilvyi* (syn. *G. castaneae*) and *G. daii* (Crous et al. 2012; Jiang and Tian 2019). *Gnomoniopsis smithogilvyi* and *G. castaneae* were proposed by two independent studies, from rotten fruits of *Castanea sativa* (Crous et al. 2012; Visentin et al. 2012). However, Shuttleworth et al. (2015) proved that *Gnomoniopsis smithogilvyi* and *G. castaneae* are conspecific based on a comparative morphological analysis and five-marker phylogenetic analysis. The fungal name *Gnomoniopsis smithogilvyi* was published earlier than *G. castaneae*, hence *G. smithogilvyi* has priority over *G. castaneae*.

*Gnomoniopsis smithogilvyi* is an important nut rot agent on chestnut nuts, an endophyte in asymptomatic flowers, leaves and stems, and a saprobe on dead burrs and branches (Crous et al. 2012; Visentin et al. 2012). Moreover, this species has been reported as a severe bark pathogen on *Castanea* in several countries (Dar and Rai 2013, 2015; Pasche et al. 2016; Lewis et al. 2017; Trapiello et al. 2018; Lione et al. 2019). In China, *Gnomoniopsis* from rotten Chinese chestnut has proved to be a different species, namely *Gnomoniopsis daii* (Jiang and Tian 2019). In this study, we focused on the symptom, taxonomy and pathogenicity aspects of *Gnomoniopsis* species from cankered tissues on Chinese chestnut trees.

## Materials and methods

### Sample collection and isolation

**Figure 1.**
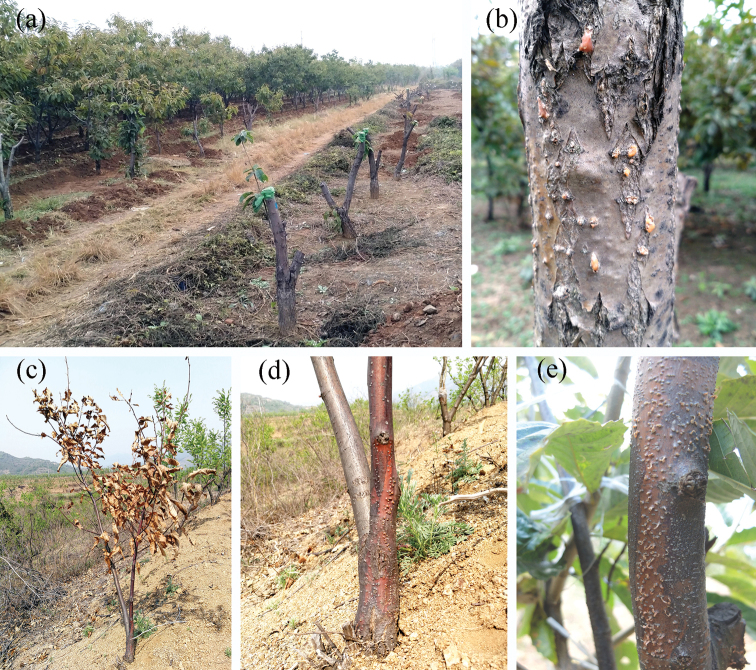
Symptoms caused by *Gnomoniopsis chinensis* on Chinese chestnut (*Castanea mollissima*) **a, b** severe cankers on adult trees **c** a dead young tree **d** lesion with conidiomata on the bark near the root **e** lesion with conidiomata on the stem.

During 2016 to 2019, investigations were conducted in chestnut plantations of nine provinces/municipalities in China, including Beijing, Fujian, Hebei, Hubei, Hunan, Liaoning, Shandong, Shaanxi and Tianjin. Typical *Gnomoniopsis* canker symptoms were only observed in Hebei Province (Fig. 1). Symptomatic barks from stems and cankered branches were collected in brown paper bags and transported to the laboratory for fungal isolations and further study. Single conidial isolates were acquired from asexual fruiting structures by removing a mucoid conidial mass from pycnidial ostioles, and spreading the suspension on the surface of potato dextrose agar (PDA; 200 g potatoes, 20 g dextrose, 20 g agar per L). Agar plates were incubated at 25 °C to induce germination of conidia. After inoculation for up to 36 h, single germinating conidia were then transferred to clean plates under a dissecting stereomicroscope with a sterile needle. Specimens and cultures were deposited and maintained in the Museum of Beijing Forestry University (BJFC) and China Forestry Culture Collection Center (CFCC), Beijing, China, respectively.

### DNA extraction and phylogenetic analysis

Genomic DNA was extracted from mycelium grown on PDA using a CTAB (cetyltrimethylammonium bromide) method (Doyle and Doyle 1990). Three partial loci, including the 5.8S nuclear ribosomal DNA gene with the two flanking internally transcribed spacer (ITS) regions, the translation elongation factor 1a (*tef1*), and the β-tubulin gene 2 (*tub2*), were amplified using the following primer pairs: ITS1 and ITS4 for ITS (White et al. 1990), EF1-728F and EF1-1567R for *tef1* (Carbone and Kohn 1999), and Bt2a and Bt2b for *tub2* (Glass and Donaldson 1995). The PCR conditions were: initial denaturation step of 5 min at 94 °C, followed by 35 cycles of 30 s at 94 °C, 50 s at 48 °C (ITS) or 54 °C (tef1) or 52 °C (tub2), and 1 min at 72 °C, and a final elongation step of 7 min at 72 °C. The PCR amplification products were scored visually by electrophoresis in 2 % agarose gels. The DNA sequencing was performed using an ABI PRISM 3730XL DNA Analyzer with BigDye Terminater Kit v.3.1 (Invitrogen) at the Shanghai Invitrogen Biological Technology Company Limited (Beijing, China). To assess the phylogenetic position of our isolates within the genus *Gnomoniopsis*, phylogenetic analyses were performed based on combined ITS, *tef1* and *tub2* sequence data, with *Sirococcus castaneae* (CBS 142041) and *Apiognomonia errabunda* (CBS 342.86) selected as outgroup taxa. The GenBank accession numbers of sequences used in the analysis are given in Table 1, which were aligned and edited manually in MEGA6 (Tamura et al. 2013). Maximum likelihood (ML) analysis was used for phylogenetic inferences of the concatenated alignments. ML analysis was implemented on the CIPRES Science Gateway portal using RAxML-HPC BlackBox v. 8.2.10 (Stamatakis 2014).

**Table 1.** Isolates and GenBank accession numbers used in this study.

Species	Country	Host	Strain	GenBank Accession Number		
				ITS	*tub2*	*tef1*
*Apiognomonia veneta*	France	*Platanus occidentalis*	CBS 342.86	DQ313531	EU219235	DQ318036
*Gnomoniopsis alderdunensis*	USA	*Rubus pedatus*	CBS 125679	GU320826	GU320788	GU320813
*Gnomoniopsis alderdunensis*	USA	*Rubus parviflorus*	CBS 125680	GU320825	GU320787	GU320801
*Gnomoniopsis alderdunensis*	USA	*Rubus parviflorus*	CBS 125681	GU320827	GU320789	GU320802
*Gnomoniopsis chamaemori*	Finland	*Rubus chamaemorus*	CBS 804.79	GU320817	GU320777	GU320809
***Gnomoniopsis chinensis***	**China**	***Castanea mollissima***	**CFCC 52286**	**MG866032**	**MH545366**	**MH545370**
***Gnomoniopsis chinensis***	**China**	***Castanea mollissima***	**CFCC 52287**	**MG866033**	**MH545367**	**MH545371**
***Gnomoniopsis chinensis***	**China**	***Castanea mollissima***	**CFCC 52288**	**MG866034**	**MH545368**	**MH545372**
***Gnomoniopsis chinensis***	**China**	***Castanea mollissima***	**CFCC 52289**	**MG866035**	**MH545369**	**MH545373**
*Gnomoniopsis clavulata*	USA	*Quercus falcata*	CBS 121255	EU254818	EU219211	GU320807
*Gnomoniopsis comari*	Finland	*Comarum palustre*	CBS 806.79	EU254821	EU219156	GU320810
*Gnomoniopsis comari*	Finland	*Comarum palustre*	CBS 807.79	EU254822	GU320779	GU320814
*Gnomoniopsis comari*	Switzerland	*Comarum palustre*	CBS 809.79	EU254823	GU320778	GU320794
*Gnomoniopsis daii*	China	*Castanea mollissima*	CFCC 54043	MN598671	MN605517	MN605519
*Gnomoniopsis daii*	China	*Castanea mollissima*	CMF002B	MN598672	MN605518	MN605520
*Gnomoniopsis fructicola*	USA	*Fragaria vesca*	CBS 121226	EU254824	EU219144	GU320792
*Gnomoniopsis fructicola*	France	*Fragaria* sp.	CBS 208.34	EU254826	EU219149	GU320808
*Gnomoniopsis fructicola*	USA	*Fragaria* sp.	CBS 125671	GU320816	GU320776	GU320793
*Gnomoniopsis guttulata*	Bulgaria	*Agrimonia eupatoria*	MS 0312	EU254812	NA	NA
*Gnomoniopsis idaeicola*	USA	*Rubus* sp.	CBS 125672	GU320823	GU320781	GU320797
*Gnomoniopsis idaeicola*	USA	*Rubus pedatus*	CBS 125673	GU320824	GU320782	GU320798
*Gnomoniopsis idaeicola*	France	*Rubus* sp.	CBS 125674	GU320820	GU320780	GU320796
*Gnomoniopsis idaeicola*	USA	*Rubus procerus*	CBS 125675	GU320822	GU320783	GU320799
*Gnomoniopsis idaeicola*	USA	*Rubus procerus*	CBS 125676	GU320821	GU320784	GU320811
*Gnomoniopsis macounii*	USA	*Spiraea* sp.	CBS 121468	EU254762	EU219126	GU320804
*Gnomoniopsis occulta*	USA	*Potentilla* sp.	CBS 125677	GU320828	GU320785	GU320812
*Gnomoniopsis occulta*	USA	*Potentilla* sp.	CBS 125678	GU320829	GU320786	GU320800
*Gnomoniopsis paraclavulata*	USA	*Quercus alba*	CBS 123202	GU320830	GU320775	GU320815
*Gnomoniopsis racemula*	USA	*Chamerion angustifolium*	CBS 121469	EU254841	EU219125	GU320803
*Gnomoniopsis sanguisorbae*	Switzerland	*Sanguisorba minor*	CBS 858.79	GU320818	GU320790	GU320805
*Gnomoniopsis smithogilvyi*	Australia	*Castanea* sp.	CBS 130190	JQ910642	JQ910639	KR072534
*Gnomoniopsis smithogilvyi*	Australia	*Castanea* sp.	CBS 130189	JQ910644	JQ910641	KR072535
*Gnomoniopsis smithogilvyi*	Australia	*Castanea* sp.	CBS 130188	JQ910643	JQ910640	KR072536
*Gnomoniopsis smithogilvyi*	Italy	*Castanea sativa*	MUT 401	HM142946	KR072532	KR072537
*Gnomoniopsis smithogilvyi*	New Zealand	*Castanea sativa*	MUT 411	HM142948	KR072533	KR072538
*Gnomoniopsis tormentillae*	Switzerland	*Potentilla* sp.	CBS 904.79	EU254856	EU219165	GU320795
*Sirococcus castaneae*	Switzerland	*Castanea sativa*	CBS 142041	KX929744	KX958443	KX929710

Note: NA, not applicable. Strains in this study are identified in bold.

### Morphological identification and characterization

Species identification was based on morphological features of the asexual fruiting bodies produced on infected plant tissues, supplemented by cultural characteristics. Hence, cross-sections were prepared by hand using a double-edge blade. Morphological characteristics of the fruiting bodies including: size of conidiomata and locules; size and shape of conidiophores and conidia were determined under a Nikon AZ100 dissecting stereomicroscope. More than 20 fruiting bodies were sectioned, and 50 conidia were selected randomly for measurement using a Leica compound microscope (LM, DM 2500). Cultural characteristics of isolates incubated on PDA in the dark at 25 °C were recorded, including the colony color and pycnidium structures (Rayner 1970).

### Pathogenicity trials

Two isolates of *Gnomoniopsis chinensis* (ex-type strain: CFCC 52286; CFCC 52288) were used for inoculations, and agar plugs were used as the negative control. Isolates were grown on PDA for five days at 25 °C before the tests. Inoculations were performed on detached branches and 3-year-old seedlings of *Castanea mollissima*, respectively. The detached branches and young seedling were collected from Hebei Province where the disease is emerging. The healthy chestnut branches (2 cm in diameter) were sampled from an adult chestnut tree and cut into pieces of 20 cm length. A total of 30 fresh and healthy branches and 15 seedlings were used for the pathogenicity tests. Ten branches and five seedlings were inoculated with each isolate and the negative control. For incubations, incisions were made on the middle of the detached branches and 1 cm above the midpoint of the seedling stem to expose the cambium using a 5-mm-diameter cork borer. Discs of agar were cut from the actively growing margins of the cultures and these were placed into wounds of the same size on the chestnut barks. Inoculated wounds and ends of inoculated branches were sealed with parafilm to reduce desiccation and the chance of contamination. The tested seedlings and branch segments were maintained in the greenhouse randomly at 25 °C under natural light conditions. Detached branches were inoculated in November 2017, and the young seedlings were tested in July 2019. The results from detached branches were evaluated after one month, and seedlings after three months, by measuring the lengths of the lesions on the cambium. The re-isolations were made from the resultant lesions from all tested branches and seedlings by cutting small pieces of discolored xylem and placing them onto the PDA plates. Re-isolations were identified based on morphology on PDA and ITS sequences. Differences among isolates in lesion length were analyzed by one-way analysis of variance (ANOVA) followed by least significant difference (LSD) tests. Statistical analysis was carried out by R software (version 3.4.3.) and considered as significant at p < 0.05.

## Results

### Phylogenetic analyses

**Figure 2.**
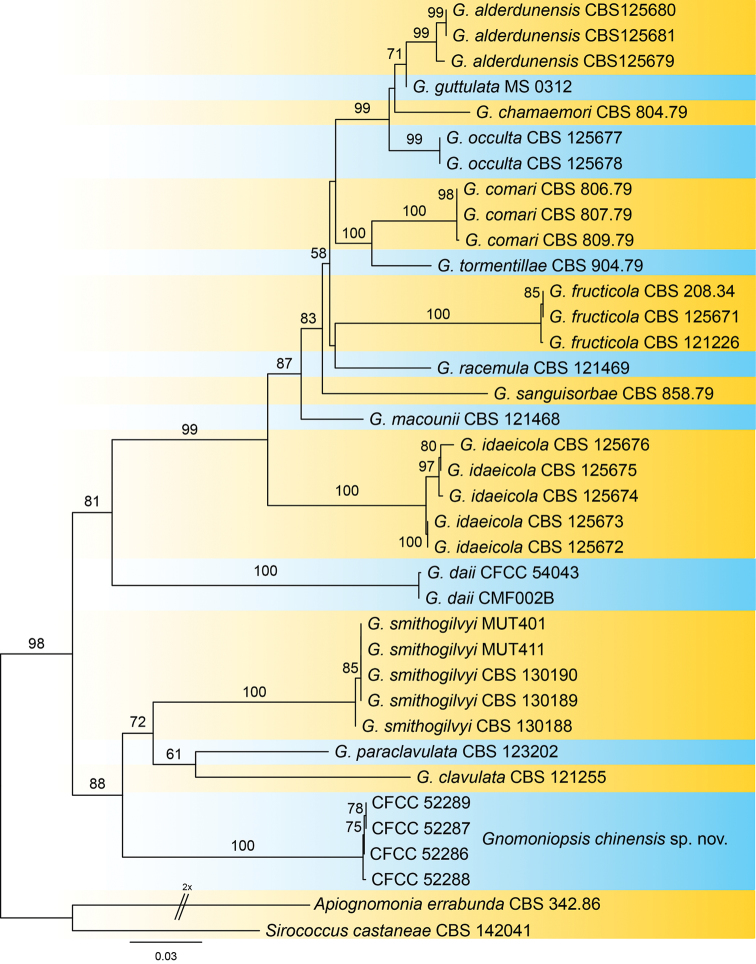
Consensus tree resulting from a RAxML analysis of combined ITS, *tef1* and *tub2* sequence alignment for species of *Gnomoniopsis*. The scale bar represents the expected number of changes per site.

The final combined ITS-*tef1*-*tub2* matrix of *Gnomoniopsis* included 35 ingroup and two outgroup taxa, comprising 1364 alignment characters. Of these, 783 characters were constant, 117 variable characters were parsimony-uninformative and 464 characters were parsimony informative. The phylogenetic tree obtained from ML analysis is shown in Figure 2, indicating that all isolates from the present study are phylogenetically different from other known species with 100% ML bootstrap support.

### Taxonomy

#### Gnomoniopsis chinensis

Taxon classificationFungiDiaporthalesGnomoniaceae

C.M. Tian & N. Jiang sp. nov.

46D7E8AA-A999-5F6D-9271-AEE0EB0B62B6

823868

Figures 3 , 4

##### Etymology.

Named after the country where it was first collected.

##### Description.

Pathogenic on stems and branches of *Castanea mollissima*. Conidiomata pseudostromatic, globose to pulvinate, occurring separately, yellow to orange, semi-immersed in bark, 400–1000 µm high, 500–1500 µm diam, unilocular, single ostiolate, forming long, wide orange tendrils, 1500–2000 µm × 400–500 µm. Conidiophores indistinct, often reduced to conidiogenous cells. Conidiogenous cells oval, hyaline, 1-celled, 6–12 µm. Conidia oval, oblate, fusiform, straight to curved, hyaline, 2–3 guttules, (6.0–)6.5–8.5(–9.0) × (2.2–)2.7–3(–3.5) µm (mean = 7.5 × 2.7 µm).

##### Culture characters.

Colonies on PDA attaining 90 mm after 20 days at 25 °C, flat, velutinous to shortly woolly, dark brown in center, gradually lightening to pale grey at margin; margin diffuse; reverse of almost same colors as surface.

##### Specimens examined.

China, Hebei Province, Chengde City, chestnut plantation, 40°24'32.16"N, 117°28'56.24"E, 262 m asl, on stems and branches of *Castanea mollissima*, Ning Jiang, 11 October 2017 (BJFC-S1380, holotype; ex-type culture, CFCC 52286). Hebei Province, Qinhuangdao City, chestnut plantation, 40°22'52.32"N, 119°11'52.18"E, 246 m asl, on branches and twigs of *Castanea mollissima*, Ning Jiang, 14 October 2017 (BJFC-S1382, paratype; living culture, CFCC 52288). Hebei Province, Tangshan City, chestnut plantation, 40°12'59.76"N, 117°59'7.24"E, 67 m asl, on stems and branches of *Castanea mollissima*, Ning Jiang, 18 October 2017 (BJFC-S1383; living culture, CFCC 52289).

##### Notes.

Three *Gnomoniopsis* species have been discovered from the host genus *Castanea*. They share similar conidial dimension (6.0–9.0 × 2.2–3.5 µm in *Gnomoniopsis chinensis* vs. 5.0–8.0 × 2.0–3.5 µm in *G. daii* vs. 6.0–9.5 × 2.0–4.0 µm in *G. smithogilvyi*) (Crous et al. 2012; Jiang and Tian 2019). However, we can distinguish them easily by the phylogram of ITS, *tef1* and *tub2* (Fig. 2). In addition, *Gnomoniopsis chinensis* and *G. daii* inhabit the Chinese chestnut (*Castanea mollissima*), but *G. smithogilvyi* on the European chestnut (*C. sativa*) and *C. crenata* × *C. sativa* hybrids.

**Figure 3.**
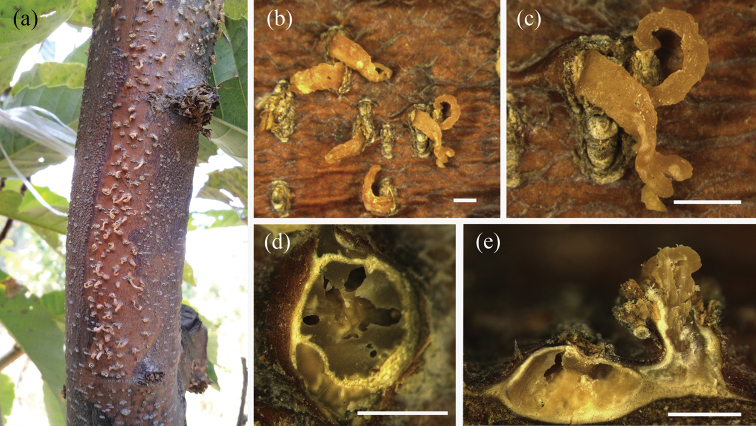
Conidiomata of *Gnomoniopsis chinensis* from *Castanea mollissima* (BJFC-S1380, holotype) **a–c** habit of conidiomata on the chestnut stem **d** transverse sections through conidiomata **e** longitudinal sections through conidiomata. Scale bars: 1 mm (**b–e**).

**Figure 4.**
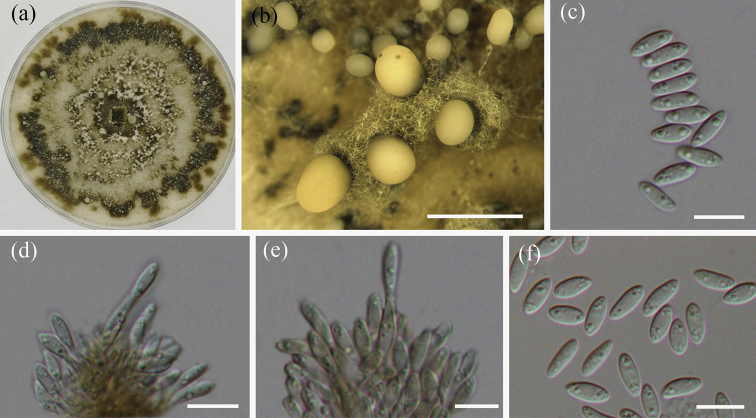
Morphology of *Gnomoniopsis chinensis* from PDA (CFCC 52286, ex-type culture) **a** colonies on PDA **b** conidiomata formed on PDA **c, f** conidia **d, e** conidiogenous cells. Scale bars: 1 mm (**b**); 10 μm (**c–f**).

### Pathogenicity trials

**Figure 5.**
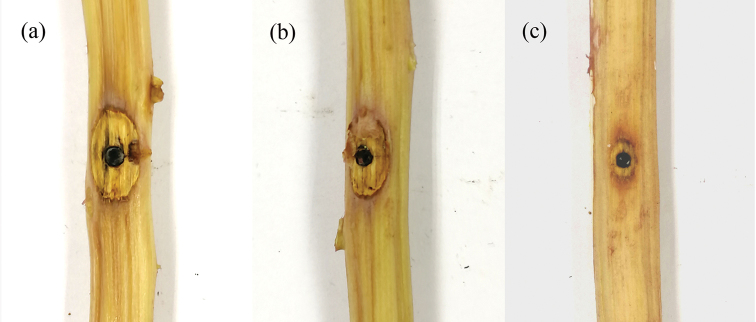
Lesions resulting from inoculation of *Gnomoniopsis chinensis* onto detached *Castanea mollissima* branches, and wound response on the negative control **a**CFCC 52288 **b**CFCC 52286 **c** negative control.

One month after inoculation on detached branches, the two *Gnomoniopsis chinensis* isolates produced lesions in the cambium of detached chestnut branches. In contrast, there was no lesion development in any of the negative control inoculations (Fig. 5). The lesion size of the two *Gnomoniopsis chinensis* isolates (CFCC 52286 and CFCC 52288) showed no significantly difference, while both of them were significantly longer than the negative control (P < 0.05) . *Gnomoniopsis chinensis* was consistently re-isolated from lesions.

**Figure 6.**
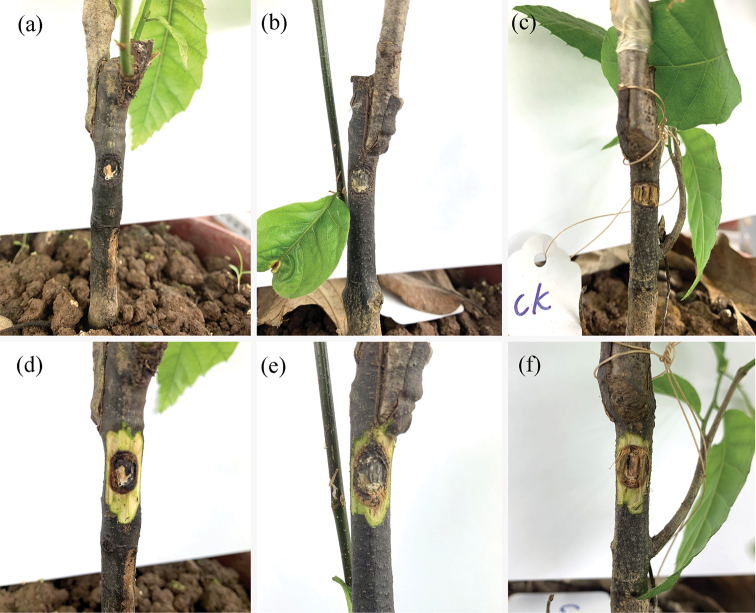
Lesions resulting from inoculation of *Gnomoniopsis chinensis* onto 3-year-old *Castanea mollissima* seedlings, and wound response on the negative control **a, d**CFCC 52288 **b, e**CFCC 52286 **c, f** negative control. Row 1: lesions on the bark; row 2: lesions beneath the bark.

Three months after inoculation on young seedlings, two isolates *Gnomoniopsis chinensis* and the negative control, produced minor lesions (Fig. 6). Statistical analyses of data showed no significant difference among two isolates *Gnomoniopsis chinensis* and the negative control (P < 0.05). However, *Gnomoniopsis chinensis* was still re-isolated successfully from the minor lesions caused by CFCC 52286 and CFCC 52288 and not from the negative control inoculations.

## Discussion

In the past years, our team focused on the fungi inhabiting Chinese chestnut (*Castanea mollissima*) trees from their taxonomy and pathogenicity aspects. Several fungi including *Aurantiosacculus castaneae*, *Cryphonectria neoparasitica*, *Cry. parasitica*, *Endothia chinensis* and *Gnomoniopsis daii* have been proven to cause branch canker or fruit rot (Jiang et al. 2019b; Jiang and Tian 2019). Other fungi were reported to be associated with branch canker, however, they were not confirmed by incubation tests, including *Aplosporella javeedii*, *Coryneum gigasporum*, *Co. sinense*, *Co. suttonii*, *Co. umbonatum*, *Cytospora ceratospermopsis*, *Cy. kuanchengensis*, *Cy. leucostoma*, *Cy. myrtagena*, *Cy. Schulzeri*, *Cy. xinglongensis*, *Dendrostoma aurorae*, *Den. castaneae*, *Den. castaneicola*, *Den. chinense*, *Den. parasiticum*, *Den. shaanxiense*, *Den. shandongense*, *Lopadostoma americanum*, *Melanops castaneicola*, *Myrmaecium fulvopruinatum*, *Neopseudomelanconis castaneae* (Jiang et al. 2018b, c, d, 2019a, 2020). Subsequently, *Dendrostoma atlanticum* and *Den. castaneum* were reported from European chestnut (*Castanea sativa*) trees (Jaklitsch and Voglmayr 2019). Different *Dendrostoma* species were discovered from the Chinese and European chestnut stems, branches and twigs, which indicates similar plant and fungi interactions in different continents. Another example is that *Gnomoniopsis daii* causes Chinese chestnut rot and *Gnomoniopsis smithogilvyi* causes European chestnut rot (Crous et al. 2012; Jiang and Tian 2019). Interestingly, this study reveals a novel *Gnomoniopsis* species, *G. chinensis*, as an opportunistic pathogen causing bark cankers on Chinese chestnut, which is different from *Gnomoniopsis smithogilvyi* causing both nut rot and bark cankers (Crous et al. 2012; Visentin et al. 2012; Dar and Rai 2013, 2015; Pasche et al. 2016; Lewis et al. 2017; Trapiello et al. 2018).

*Gnomoniopsis* species appear host-specific, inhabiting hosts of three families, viz. Betulaceae, Fagaceae, Rosaceae and Onagraceae (Sogonov et al. 2008; Walker et al. 2010; Visentin et al. 2012; Linaldeddu et al. 2016). Five species have been discovered from fagaceous hosts, and they are similar in conidial size (Table 2). *Gnomoniopsis clavulata* and *G. paraclavulata* were recorded on *Fagus* or *Quercus* trees (Sogonov et al. 2008). *Gnomoniopsis chinensis* and *G. daii* were discovered only from *Castanea* trees. It is hard to distinguish them by the currently known conidial characteristics. However, all currently known *Gnomoniopsis* species can be successfully distinguished by phylogenetic analysis based on ITS, *tef1* and *tub2*.

**Table 2.** Conidial size of *Gnomoniopsis* species from fagaceous hosts.

**Species**	**Conidial length (µm)**	**Conidial width (µm)**	**Reference**
*Gnomoniopsis chinensis*	(6.0–)6.5–8.5(–9.0)	(2.2–)2.7–3(–3.5)	This study
*Gnomoniopsis clavulata*	(5–)6–6.5(–8)	(2–)2.5–3(–4)	Sogonov et al. 2008
*Gnomoniopsis daii*	(5.0–)5.5–7.0(–8.0)	2.0–3.5	Jiang and Tian 2019
*Gnomoniopsis paraclavulata*	(6–)7.5–8(–9.5)	(2–)3–3(–3.5)	Sogonov et al. 2008
*Gnomoniopsis smithogilvyi*	(6.0–)8(–9.5)	(2.0–)2.5(–4.0)	Crous et al. 2012

Stevanović et al. (2019) reported *Gnomoniopsis idaeicola* to cause blackberry canker and wilting in Serbia. With the same signs on the host bark, especially the wide, orange tendrils emerging from hosts’ glaucous lenticels, *Gnomoniopsis chinensis* appeared to be an emerging pathogen on *Castanea mollissima*. Chestnut blight, caused by *Cryphonectria parasitica*, a notorious bark disease on chestnut trees worldwide (Rigling and Prospero 2018), can be distinguished from chestnut *Gnomoniopsis* canker, and the presence of mycelial fans in the cambial region. Nowadays, we have characterized the two canker pathogens on Chinese and European chestnut trees, *Gnomoniopsis chinensis* and *G. smithogilvyi*. They appear not to be very pathogenic to their native hosts, but the pathogenicity to non-native hosts is still unknown. *Gnomoniopsis* and *Cryphonectria* belong to the same fungal order Diaporthales, and are similar in some aspects. Hence, more work on these two pathogens is necessary on both *Castanea mollissima* and *C. sativa*. In addition, considering the high value of the plant genus, *Castanea*, and the current situation of serious commercial loss caused by various fungi, more comprehensive and detailed investigations are necessary to understand the diversity of microbes on the hosts and their functions.

## Supplementary Material

XML Treatment for Gnomoniopsis chinensis
